# Moral distress and positive experiences of ICU staff during the COVID-19 pandemic: lessons learned

**DOI:** 10.1186/s12910-023-00919-8

**Published:** 2023-06-08

**Authors:** Mark L. van Zuylen, Janine C. de Snoo-Trimp, Suzanne Metselaar, Dave A. Dongelmans, Bert Molewijk

**Affiliations:** 1 Department of Anaesthesiology, Amsterdam UMC, University of Amsterdam, Meibergdreef, Amsterdam, The Netherlands; 2 Department of Ethics, Law and Humanities, Amsterdam UMC, VU University, De Boelelaan 1089a, Amsterdam, 1081 HV The Netherlands; 3 Department of Intensive Care, Amsterdam, UMC, University of Amsterdam, Meibergdreef, Amsterdam, The Netherlands; 4 Center of Medical Ethics, Institute of Health and Society, University of Oslo, Oslo, Norway

**Keywords:** COVID-19, Intensive care, Critical care, Multidisciplinary, Medical ethics, Moral challenges, Moral distress, Ethical climate, Moral resilience, Ethics support services

## Abstract

**Background:**

The COVID-19 pandemic causes moral challenges and moral distress for healthcare professionals and, due to an increased work load, reduces time and opportunities for clinical ethics support services. Nevertheless, healthcare professionals could also identify essential elements to maintain or change in the future, as moral distress and moral challenges can indicate opportunities to strengthen moral resilience of healthcare professionals and organisations.

This study describes 1) the experienced moral distress, challenges and ethical climate concerning end-of-life care of Intensive Care Unit staff during the first wave of the COVID-19 pandemic and 2) their positive experiences and lessons learned, which function as directions for future forms of ethics support.

**Methods:**

A cross-sectional survey combining quantitative and qualitative elements was sent to all healthcare professionals who worked at the Intensive Care Unit of the Amsterdam UMC - Location AMC during the first wave of the COVID-19 pandemic. The survey consisted of 36 items about moral distress (concerning quality of care and emotional stress), team cooperation, ethical climate and (ways of dealing with) end-of-life decisions, and two open questions about positive experiences and suggestions for work improvement.

**Results:**

All 178 respondents (response rate: 25–32%) showed signs of moral distress, and experienced moral dilemmas in end-of-life decisions, whereas they experienced a relatively positive ethical climate. Nurses scored significantly higher than physicians on most items. Positive experiences were mostly related to ‘team cooperation’, ‘team solidarity’ and ‘work ethic’. Lessons learned were mostly related to ‘quality of care’ and ‘professional qualities’.

**Conclusions:**

Despite the crisis, positive experiences related to ethical climate, team members and overall work ethic were reported by Intensive Care Unit staff and quality and organisation of care lessons were learned. Ethics support services can be tailored to reflect on morally challenging situations, restore moral resilience, create space for self-care and strengthen team spirit. This can improve healthcare professionals’ dealing of inherent moral challenges and moral distress in order to strengthen both individual and organisational moral resilience.

**Trial registration:**

The trial was registered on The Netherlands Trial Register, number NL9177.

**Supplementary Information:**

The online version contains supplementary material available at 10.1186/s12910-023-00919-8.

## Background

The COVID-19 pandemic caused a worldwide crisis and led to extreme working conditions for Intensive Care Unit (ICU) staff worldwide, causing many moral challenges and little time for the use of ethics support services such as ethics consultation and moral case deliberations [1–3]. The sheer amount of critically ill patients caused a surge in workload at the ICU [4, 5], often resulting in a decreased nurse-to-patient ratio and longer shifts than usual [6]. The influx of COVID patients at the ICU and their disease severity also caused various moral challenges related to the impossibility of family visits of critically ill family members, a subsequent reduced quality of end-of-life (EOL) support [7, 8], the threat of a possible triage [9, 10] and concerns for care workers’ own health and safety [11]. Because ICU’s were facing a new disease, little was known about how patients with COVID-19 would recover over time. This uncertainty about prognosis resulted in even more complicated EOL decision-making than usual.

High workload and the experience of severe moral challenges can contribute to an increased level of moral distress amongst ICU staff [12–19]. Moral challenges are challenges arising in situations of uncertainty about the right course of action or situations of conflicting values and principles, either intrapersonal or interpersonal [20, 21]. Moral distress has been defined as negative feelings such as sadness, powerlessness, frustration and regret resulting from experiencing a moral event [22] and is characterized by ‘the perception of being morally compromised for not being able to be oneself in a situation in which you feel that you should (but were not) able to do the right thing’ [12, 23].

Moral distress may result in increased fatigue and decreased job satisfaction, higher turnover rates, sick leave and burnout [13–19]. It may even lead to enduring feelings of shame, regret, self-doubt and guilt [24], also defined as ‘moral injury’ [25]. Generally, nursing staff have a higher incidence of moral distress than physicians, often attributed to the fact that nurses regularly feel that they are not sufficiently involved in discussions and decision-making processes about ethically complex situations, whilst at the same time having to perform morally critical actions based on decisions made by others [26, 27]. This feeling of being insufficiently involved in decision-making processes has indeed recently been confirmed in a survey on experiences during the COVID-19 crisis among Spanish ICU nurses [28].

However, when a healthcare professional experiences moral distress, it is not exclusively something negative, as it shows that they are morally involved. It may lead to reflection on one’s own actions and integrity, can stimulate creative and innovative solutions contributing to the quality of care, and in the end may result in a better mental health and stronger moral resilience [29]. Moral resilience refers to the capacity ‘to restore or sustain integrity in response to moral adversity’ [14]. To enhance and preserve moral resilience, an open organisational and supportive team environment, providing spaces for healthcare staff to jointly reflect upon their moral challenges and moral dilemmas is crucial [12, 25]. Studies have shown that ethics support services can contribute to a good team cooperation and a positive ethical climate which subsequently can contribute to a decreased level of moral distress [22, 30, 31] and may help in alleviating negative consequences often associated with moral distress (e.g. sick leave, burnout) [32]. Ethics support acknowledges the inherent moral ambiguity and uncertainty associated with moral challenges and offers ways of dealing with the sometimes inherently tragic dimensions in unideal healthcare practices. Ethics support services have therefore been strongly suggested as a way to ‘identify and untangle the complex ethical issues that cause moral distress and help mitigate the negative effects of such distress’ [11, 25].

This study aimed to describe experiences from the multidisciplinary ICU staff during the first wave of the COVID-19 pandemic, with two research questions: 1) What did they experience regarding moral distress, the quality of team cooperation, the ethical climate and (dealing with) moral challenges surrounding EOL decisions? And 2) What positive experiences and suggestions for work improvement did they encounter (if any)? The second concerns something which rarely gets attention in moral distress or COVID-19 studies. Thirdly, we wanted to investigate whether there were any differences in the aforementioned experiences between nursing staff and physicians. This study will provide insight into elements that can be maintained at the ICU, and the lessons learned; both for future COVID-19 waves, as for the new normal at our ICU’s.

## Methods

### Design and setting

This was a cross-sectional, single-centre questionnaire study, performed in the Amsterdam UMC – Location AMC, a tertiary referral hospital in Amsterdam, the Netherlands. During the first wave of the COVID-19 pandemic the ICU could hold a total of 32 ventilated COVID-19 patients and 8 regular ICU patients. A total of 88 patients were admitted at the ICU between March and May 2020. At the height of the first wave of COVID-19 in the Netherlands, a maximum of 1428 patients with COVID-19 were admitted to ICUs across the country.

### Study site support

At the start of the pandemic, the possible moral challenges and related moral stress that ICU staff might experience were acknowledged: daily, two debriefings led by medical psychologists were held to discuss any possible work difficulties or challenges staff had faced.

During the first wave of the COVID-19 crisis, staff from other departments who stepped in to help were linked to ICU staff through a buddy system. This way they could get used to the new department in a more safe and secure way. At the beginning of the first wave shift duration was shortened, from 8–12 h to a maximum of 8 h. Initially, due to the shortage of personal protective equipment, staff could only break once per shift. However, as soon as enough personal protective equipment was available, this was increased to two breaks per shift. Throughout the first wave free food and drinks were provided for the entire ICU staff during break time.

A 24/7 ethics support line, as part of a general 24/7 support line, was also set up by the department of Ethics, Law & Humanities to provide additional ethical support: ICU staff who were morally troubled by what they experienced could call for ad hoc ethics consultation by telephone or further ethics support actions (e.g. planning a moral case deliberation with the team).

### Respondents

All employees of medical disciplines (i.e. all physicians, nurses (in training), nurse anaesthetists (in training), surgical assistants and others performing ancillary tasks) who *possibly* had worked at the Intensive Care department of the Amsterdam UMC - Location AMC during the first wave of the COVID-19 crisis received an invitation by email to fill out the questionnaire online. Some automated mailing lists were used, thus reaching relatively more people than those who actually worked during the first wave. The questionnaire was sent on August 20, 2020 and a reminder email was sent on October 6, 2020.

### Questionnaire

To assess moral distress, ethical climate and moral challenges concerning end-of-life care during the first wave of COVID-19 on the ICU, the authors MvZ, JS and BM^1^ reviewed several pre-existing questionnaires for suitable questions. In the event questions were not chosen unanimously, the final decision was made by consensus. The multiple choice section of the questionnaire was then comprised of selected questions from the 32-item ethical decision-making climate questionnaire [33] (EDMCQ), a Dutch version of the revised 21-item moral distress scale (MDS-R) [34] and the Belgian Individual Detection and Reflection Tool for Moral Stress [35] which used items from other moral distress scales including the MDS-R [34, 36, 37]. Details about (and reasons for selecting) these questionnaires are described in Supplementary file 1. The initial questionnaire was reviewed by a psycho-metric expert and piloted among some ICU staff.

The final questionnaire consisted of 36 multiple choice questions, and 2 open-ended questions about positive experiences (‘*What positive things are worth preserving in the future?’*) and suggestions for work improvement (‘*Are there things you would do differently if there was a similar period in the future?*’). The 36 questions were subdivided into 6 sections: moral distress – quality of care (*n* = 10), moral distress– emotional stress (*n* = 7), team cooperation (*n* = 5), ethical climate (*n* = 3), ways of dealing with challenges around EOL decisions (*n* = 11). A 5-point Likert scale was used for all questions, ranging from ‘totally agree’ to ‘totally disagree’.

### Ethical considerations

Ethics approval was obtained from the local medical ethics committee of the Amsterdam UMC - location AMC (METC AMC, The Netherlands; reference number AMC W20_361 # 20.401). The questionnaire was send anonymously via email and respondents could decide for themselves if they wanted to participate. The study was performed according to the Declaration of Helsinki [38]. The trial was registered on The Netherlands Trial Register, number NL9177 [39]. Performance, recording, analysis and reporting was done according to the Strengthening the Reporting of Observational Studies in Epidemiology (STROBE) Statement for reporting observational studies [40].

### Data analysis

We aimed to extensively map the moral challenges and positive experiences that staff at the Intensive Care Unit of the Amsterdam UMC - Location AMC mentioned with regard to the first wave of the COVID-19 crisis. Furthermore, we assessed experienced moral distress, ethical climate and what, if any, ICU staff wanted to change in the event of a second wave. Lastly, we analysed whether there were any differences between the groups of doctors and nurses regarding the aforementioned. All quantitative and qualitative data were managed using Castor Electronic Data Capture (EDC) [41].

#### Quantitative analysis

Answers for multiple choice questions were assessed and frequency distributions were visualized per subsection. Inter-item correlations were calculated with Exploratory Factor Analysis to check how items fitted with other items within the predefined subsection. Because of the ordinal data, Mann-Whitney U tests were used to establish any differences between doctors and nursing staff with regards to moral dilemmas and moral issues. All statistical analyses were performed using SPSS software (version 26.0; IBM Corp., Armonk, New York, Unites Stated of America) [42].

#### Qualitative analysis of answers to the two open-ended questions

Content analysis according to the framework method was applied to the answers to the two open-ended questions in order to systematically code and categorise qualitative data [43]. The framework method is commonly used in analysing open-ended questions in surveys, when the study purpose concerns an inquiry of *content* (e.g., the ‘what’-question), instead of an interest in the *amount*, range or priority of answers (e.g., the ‘how many’-question). It is considered as especially useful when assessing experiences in a transparent manner [44, 45]. Using an inductive approach, all meaningful fragments in the open answers (‘meaningful units’) were coded and grouped by MvZ, JS and BM into categories and subcategories around similar and interrelated concepts. Later, the (sub)categories were grouped into clusters (e.g. ‘team’). To control for the subjective and interpretative process, MvZ, JS and BM reviewed all clusters and (sub)categories during regular discussions (four phases) ultimately leading to a consensus of the final framework (i.e. the coding tree). Finally, to inform the reader about the proportion of responses for each cluster, their number of meaningful units was also presented.

## Results

### Response rate and demographics

A total of 178 employees filled out the questionnaire and were included in the present analysis. Because some automated mailing lists were used, the questionnaire was sent to 714 employees, yet approximately 550 of them actually worked at the ICU during the first wave, indicating a response rate of 25–32 percent. From the 178 respondents, 129 and 132 respondents also answered the first, respectively second, open questions about positive experiences and things that should change during a possible second wave. Answers to the two open-ended questions consisted of approximately 10 words, ranging from one or a few words to one or more sentences. A total of respectively 240 and 248 meaningful units emerged from the answers to the first and second open-ended question.

Demographic data are presented in Table 1. 132 (74%) respondents were female, 48 (27%) were physicians performing ICU tasks (21 of whom were already employed at the ICU), 99 (56%) were nurses performing ICU tasks (41 of whom were already employed at the ICU) and 31 (17%) were healthcare professionals performing ancillary tasks.

**Table 1:** Demographic data

**Respondents questionnaire**	**Total*** n* (%)
**Respondents**	
Total	178 (100)
**Gender**	
Female	132 (74)
Male	46 (26)
**Profession** *n* (%)	
Intensivist^c^	8 (5)
ICU Nurse^d^	37 (21)
Fellow Intensive Care^c^	3 (2)
Specialist registrar ICU^c^	8 (5)
ICU Nurse in training^d^	4 (2)
Senior house officer ICU^c^	2 (1)
Medical specialist non-ICU^c^	5 (3)
Specialist registrar non-ICU^c^	22 (12)
Nurse anaesthetist^d^	20 (11)
Nurse non-ICU^d^	38 (21)
Surgical assistant	13 (7)
Doctor performing ancillary tasks^a^	8 (5)
Other performing ancillary tasks^b^	10 (6)
**Professional experience**	
0–2 years	42 (24)
3–5 years	37 (21)
6–10 years	31 (17)
11–15 years	16 (9)
>15 years	52 (29)

^a^Including medical specialists, researchers and senior house officers

^b^Including physiotherapists, nurses and psychologists

^c^Physicians who performed ICU tasks, excluding ancillary tasks

^d^Nurses who performed ICU tasks, excluding ancillary tasks

### Questionnaire responses

Quantitative data for all respondents are presented in Table 2. Comparison of answers from physicians performing ICU tasks versus nurses performing ICU tasks are presented in Table 3. Inter-item correlations showed acceptable associations between the items in each category (mean = 0.29; SD = 0.01; 95% CI = 0.27–0.31).

**Table 2:** Answers^a^ to questionnaire (all respondents)

**Questions**	**Total** **(*n*) %**	**Agree**^b^ **(n) %**		**Disagree**^c^ **(n) %**	
Moral distress - Quality of care					
*1. I felt I delivered the same quality of care compared to before.*	(178) 100		(46) 26		(100) 56
*2. It touched me to see when a patient was not receiving good care.*	(178) 100		(112) 63		(20) 11
*3. In most situations I had a strong sense of what did not constitute good care.*	(178) 100		(116) 65		(13) 7
*4. I had strong beliefs about ‘good’ and ‘bad’ patient care.*	(178) 100		(123) 69		(12) 7
*5. I feel my colleagues provided good care.*	(178) 100		(126) 71		(11) 6
6. I felt I carried out medical tests and treatments which I myself found unnecessary.	(178) 100		(23) 13		(126) 71
7. I witnessed a patient suffering as a result of a lack of continuity of caregivers.	(178) 100		(38) 21		(108) 61
8. I felt had to choose between good care and something else I find important.	(178) 100		(52) 29		(86) 48
9. I felt we provided suboptimal care because there was not enough personal protective equipment, time or manpower available.	(178) 100		(76) 43		(82) 46
10. I felt I could do less for the patients than I used to.	(178) 100		(100) 56		(58) 33
Moral distress – Emotional stress					
*11. Strong feelings arose when I saw a patient suffering.*	(178) 100	(87) 49		(40) 22	
*12. I felt strongly about the well-being of the patients.*	(178) 100	(143) 80		(4) 2	
13. I felt that, in order to be able to finish my tasks, I had to put my values and views regarding good care aside.	(178) 100	(63) 35		(88) 49	
14. I was worried my work was emotionally numbing me.	(178) 100	(65) 37		(82) 46	
15. I frequently thought to myself: what am I actually doing here?	(178) 100	(73) 41		(73) 41	
16. Compared to before, I enjoyed my work less.	(178) 100	(77) 43		(70) 39	
17. I worried about my work.	(178) 100	(87) 49		(57) 32	
Team cooperation					
*1. At the ICU, there was regular reflection on the quality of care we provided from the different perspectives of the employees.*	(170) 95.6	(53) 31		(73) 43	
*2. At the ICU there was an open and constructive culture in which criticism could easily be expressed.*	(170) 95.6	(63) 37		(48) 28	
*3. At the ICU there was regular structural discussion between the various disciplines within the team about patient care.*	(170) 95.6	(69) 41		(46) 27	
*4. At the ICU there were regular opportunities for open and informal discussions between care providers.*	(170) 95.6	(99) 58		(32) 19	
*5. At the ICU, I had confidence in the professional competencies of my team members.*	(178) 100	(132) 74		(16) 9	
Ethical climate					
*1. At the ICU I was always considered and addressed as a full member of the team by everyone in the team.*	(178) 100	(109) 61		(49) 28	
*2. At the ICU, team members from another discipline respected my work.*	(178) 100	(126) 71		(26) 15	
3. I considered being vulnerable as a sign of weakness.	(170) 95.6	(27) 16		(122) 72	
Ways of dealing with challenges around end of life decisions					
*1. At the ICU there was a structured formal debrief after a difficult situation in patient care.*	(174) 97.8	(56) 32		(58) 33	
*2. At the ICU, moral and ethical problems were discussed.*	(176) 98.9	(84) 48		(40) 23	
*3. At the ICU, nurses were involved in end-of-life decisions.*	(174) 97.8	(57) 33		(33) 19	
*4. At the ICU, there was good cooperation between nurses and physicians regarding end-of-life care.*	(174) 97.8	(84) 48		(19) 11	
*5. Different opinions and values regarding end-of-life care were tolerated at the ICU.*	(177) 99.4	(104) 59		(10) 6	
*6. My colleagues understood my ideas/feelings regarding difficult end-of-life decisions.*	(177) 99.4	(86) 49		(5) 3	
7. Providing care to patients who I thought shouldn’t receive care.	(178) 100	(20) 11		(128) 72	
8. At the ICU, death was considered therapeutic failure, so decisions to scale back or not start therapy were rarely made.	(173) 97.2	(17) 10		(88) 51	
9. Starting life-saving actions that I thought only delayed death.	(178) 100	(79) 44		(50) 28	
10. At the ICU, end-of-life decisions were often postponed.	(172) 96.7	(56) 33		(49) 28	
11. At the ICU, patients with a small chance of recovery regularly occupied an ICU bed from which other patients could benefit more.	(172) 96.7	(52) 30		(37) 22	

Questions in *cursive*: question positively/neutrally formulated

Questions in non-cursive: question reversibly formulated

^a^Excluding ‘neutral’ answer

^b^Combined answers of ‘agree’ and ‘totally agree’

^c^Combined answers of ‘disagree’ and ‘totally disagree’

**Table 3:** Comparisons between physicians and nurses in answers to questionnaire^abc^

**Questions**	**Physicians** **(*n*) %**^a^	**Agree** **(n) %**^c^		**Disagree** **(n) %**^d^			**Nurses** **(*n*) %**^a^			**Agree** **(n) %**^c^				**Disagree** **(n) %**^d^		**MWU** ***p*-value**
Moral distress - Quality of care																
*1. I felt I delivered the same quality of care compared to before.*	(48) 100		(18) 38		(20) 42	(99) 100					(22) 22				(62) 63	0.013*
*2. It touched me to see when a patient was not receiving good care.*	(48) 100		(21) 44		(10) 21	(99) 100					(73) 74				(7) 7	0.001**
*3. In most situations I had a strong sense of what did not constitute good care.*	(48) 100		(29) 60		(3) 6	(99) 100					(67) 68				(9) 9	0.674
*4. I had strong beliefs about ‘good’ and ‘bad’ patient care.*	(48) 100		(28) 58		(5) 10	(99) 100					(75) 76				(5) 5	0.016*
*5. I feel my colleagues provided good care.*	(48) 100		(42) 88		(0) 0	(99) 100					(59) 60				(9) 9	0.000***
6. I felt I carried out medical tests and treatments which I myself found unnecessary.	(48) 100		(8) 17		(36) 75	(99) 100					(11) 11				(67) 68	0.208
7. I witnessed a patient suffering as a result of a lack of continuity of caregivers.	(48) 100		(10) 21		(32) 67	(99) 100					(20) 20				(56) 57	0.124
8. I felt had to choose between good care and something else I find important.	(48) 100		(5) 10		(29) 60	(99) 100					(38) 38				(43) 43	0.001**
9. I felt we provided suboptimal care because there was not enough personal protective equipment, time or manpower available.	(48) 100		(13) 27		(30) 63	(99) 100					(52) 53				(39) 39	0.001**
10. I felt I could do less for the patients than I used to.	(48) 100		(26) 54		(14) 29	(99) 100					(52) 53				(38) 38	0.662
Moral distress – Emotional stress																
*11. Strong feelings arose when I saw a patient suffering.*	(48) 100	(17) 35		(15) 31			(99) 100		(54) 55				(21) 21			0.032*
*12. I felt strongly about the well-being of the patients.*	(48) 100	(36) 75		(0) 0			(99) 100		(80) 81				(2) 2			0.768
13. I felt that, in order to be able to finish my tasks, I had to put my values and views regarding good care aside.	(48) 100	(6) 13		(32) 67			(99) 100		(51) 52				(39) 39			0.000***
14. I was worried my work was emotionally numbing me.	(48) 100	(14) 29		(26) 54			(99) 100		(41) 41				(45) 45			0.062
15. I frequently thought to myself: what am I actually doing here?	(48) 100	(13) 27		(27) 56			(99) 100		(47) 47				(35) 35			0.009**
16. Compared to before, I enjoyed my work less.	(48) 100	(11) 23		(26) 54			(99) 100		(52) 53				(34) 34			0.001**
17. I worried about my work.	(48) 100	(13) 27		(22) 46			(99) 100		(60) 61				(27) 27			0.001**
Team cooperation																
*1. At the ICU, there was regular reflection on the quality of care we provided from the different perspectives of the employees.*	(45) 94	(14) 31		(18) 40			(94) 95		(29) 31				(45) 48			0.228
*2. At the ICU there was an open and constructive culture in which criticism could easily be expressed.*	(45) 94	(27) 60		(6) 13			(94) 95		(29) 31				(35) 37			0.000***
*3. At the ICU there was regular structural discussion between the various disciplines within the team about patient care.*	(45) 94	(23) 51		(12) 27			(94) 95		(36) 38				(29) 31			0.177
*4. At the ICU there were regular opportunities for open and informal discussions between care providers.*	(45) 94	(29) 64		(4) 9			(94) 95		(55) 59				(24) 26			0.045*
*5. At the ICU, I had confidence in the professional competencies of my team members.*	(48) 100	(42) 88		(2) 4			(99) 100		(66) 67				(12) 12			0.006**
Ethical climate																
*1. At the ICU I was always considered and addressed as a full member of the team by everyone in the team.*	(48) 100	(41) 85		(3) 6			(99) 100	(57) 56				(30) 30				0.001**
*2. At the ICU, team members from another discipline respected my work.*	(48) 100	(43) 90		(1) 2			(99) 100	(68) 69				(16) 16				0.000***
3. I considered being vulnerable as a sign of weakness.	(45) 94	(5) 11		(38) 84			(94) 95	(13) 14				(70) 74				0.446
Ways of dealing with challenges around end of life decisions																
*1. At the ICU there was a structured formal debrief after a difficult situation in patient care.*	(46) 96	(17) 37		(13) 28			(98) 99	(30) 31				(35) 36				0.133
*2. At the ICU, moral and ethical problems were discussed.*	(47) 98	(30) 64		(4) 9			(99) 100	(43) 43				(31) 31				0.009**
*3. At the ICU, nurses were involved in end-of-life decisions.*	(46) 96	(19) 41		(11) 24			(98) 99	(31) 32				(20) 20				0.265
*4. At the ICU, there was good cooperation between nurses and physicians regarding end-of-life care.*	(46) 96	(31) 67		(5) 11			(98) 99	(45) 46				(12) 12				0.057
*5. Different opinions and values regarding end-of-life care were tolerated at the ICU.*	(47) 98	(40) 85		(1) 2			(99) 100	(54) 55				(7) 7				0.001**
*6. My colleagues understood my ideas/feelings regarding difficult end-of-life decisions.*	(47) 98	(33) 70		(1) 2			(99) 100	(46) 46				(4) 4				0.003**
7. Providing care to patients who I thought shouldn’t receive care.	(48) 100	(10) 21		(32) 67			(99) 100	(10) 10				(70) 71				0.504
8. At the ICU, death was considered therapeutic failure, so decisions to scale back or not start therapy were rarely made.	(46) 96	(3) 7		(36) 78			(97) 98	(11) 11				(43) 44				0.000***
9. Starting life-saving actions that I thought only delayed death.	(48) 100	(22) 46		(13) 27			(99) 100	(47) 47				(30) 30				0.793
10. At the ICU, end-of-life decisions were often postponed.	(46) 96	(15) 33		(23) 50			(96) 97	(36) 38				(20) 21				0.008**
11. At the ICU, patients with a small chance of recovery regularly occupied an ICU bed from which other patients could benefit more.	(46) 96	(11) 24		(14) 30			(96) 97	(31) 32				(17) 18				0.169

Questions in *cursive*: question positively/neutrally formulated

Questions in non-cursive: question reversibly formulated

*P*-value in bold: results deemed interesting by authors

*MWU* Mann-Whitney U test

^*^p < 0.05; **p < 0.01; ***p < 0.001

^a^Excluding ‘neutral’ answer

^b^Physicians and nurses who performed ICU tasks, excluding ancillary tasks

^c^Combined answers of ‘agree’ and ‘totally agree’

^d^Combined answers of ‘disagree’ and ‘totally disagree’

#### Moral distress

Over half of the respondents (56%) felt they did not deliver the same quality of patient care during the first wave of COVID-19 as they did before. This was true for both physicians and nurses, although it was more pronounced in the case of the nursing staff (42 vs. 63%, *p* =  < 0.001). Interestingly, 71% of all respondents felt their colleagues provided good patient care, whilst only 6% of respondents felt that their colleagues did not. Also, 56% of all respondents felt they could do less for their patients than they would normally be able to, with about half of nurses (53%) feeling this was attributable to there not being enough personal protective equipment, time or manpower available.

Most (80%) respondents still felt strongly about the well-being of their patients at the ICU. There was, however, a clear difference in the emotional stress experienced between nurses and physicians. More than a half of the nursing staff enjoyed their work less than normally, compared to fewer than a quarter of physicians (53 vs. 23%, *p* = 0.001) and 61% of nurses worried about their work, whereas just 27% of physicians worried (*p* = 0.001).

#### Team cooperation and ethical climate

Most respondents (74%) had confidence in the professional competencies of their colleagues and most respondents felt that there were regular opportunities for open and informal discussions. However, a significantly larger portion of nurses, compared to physicians, felt that an open and constructive culture in which criticism could easily be expressed was lacking at the ICU (37 vs. 13%, *p* =  < 0.001). Where 85% of physicians felt they were considered and addressed as a full member of the ICU team by everyone in the team, 30% of nurses felt they did not (*p* =  < 0.001). Nevertheless, the vast majority of respondents (71%) felt that team members from other disciplines respected their work.

#### (Ways of dealing with) moral challenges around end of life decisions

Some healthcare professionals (11%) felt that they provided care to patients who shouldn’t receive care or felt that death was considered as therapeutic failure (10%). Almost half of respondents (44%) had experienced medical interventions being started even though they felt they would only delay imminent death. One third of respondents (33%) felt that EOL decisions were often postponed. Most (59%) felt that different opinions and values regarding EOL care were tolerated at the ICU and only 3% felt that colleagues did not understood their ideas or feelings regarding difficult EOL decisions. Interestingly, physicians often (64%) felt that moral and ethical problems were discussed, with only a small portion of physicians disagreeing (9%), which is significantly different from nursing staff (43% agree and 31% disagree, *p* = 0.009).

### Positive things worth preserving in the future

**Fig. 1:**
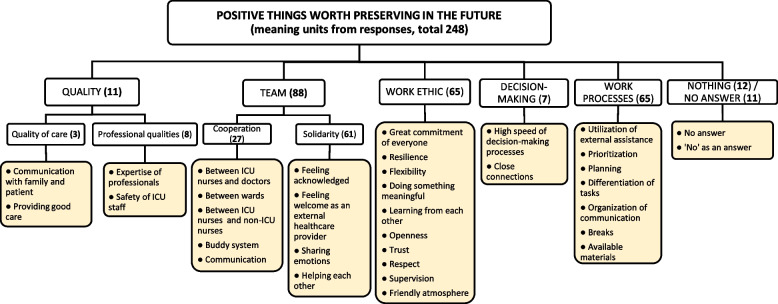
Categorization of answers to the open question ‘*What positive things are worth preserving in the future?*’. This Framework shows the overall clusters (upper level), themes and codes (last level) that emerged from analyzing the first open-ended question. The number of meaning units (total 248) is mentioned between brackets for every cluster. ICU = Intensive Care Unit

Figure 1 presents an overview of positive experiences (i.e. things that respondents would like to see preserved in the future), as described in their answers to the open-ended questions. These experiences are related to five clusters: ‘quality’, ‘team’, ‘work ethic’, ‘decision-making’, ‘work processes’. When considering the number of responses, the cluster ‘team’ was dominant, while also ‘work ethic’ and ‘work processes’ covered the majority of responses.

The first cluster, ‘quality’, consists of the subcategories ‘quality of care’ and ‘professional qualities’. Comparable to the quantitative findings, respondents often emphasized the professional qualities of their colleagues. This concerned both colleagues at the ICU as those from other departments: *‘the [external] helpers were awesome’* by also respecting their limitations: ‘*the external helpers were not asked to do things for which they were not qualified’*.

Secondly, and most prominently, experiences were focused on the cluster ‘team’, with the subcategories ‘solidarity’ and ‘cooperation’. For instance, one respondent explained the experienced solidarity and teamwork by ‘*the overall shared feeling of putting our shoulders to the wheel*’. Furthermore, respondents appreciated the supportive teamwork and room to share emotions, as one mentioned that *‘emotions were seen: there was a lot of attention for powerlessness, anger and frustrations’*. Another respondent experienced that *‘despite working so hard, there still was energy and effort to evaluate the day and give each other compliments’*. Also, respondents from non-ICU departments who entered the ICU department also mentioned to feel welcome: ‘*I was well supported when I started working there’* and *‘they took the time to explain to me their way of working’*.

Another clearly emerging cluster of positive experiences referred to the ‘work ethic’ of everyone. One respondent mentioned that *‘everyone did everything possible’* and another one expressed an *‘enormous respect for everyone’s effort*’. The flexibility and ability to deal with constantly changing circumstances, of both their direct colleagues as well as the hospital in general, was repeatedly mentioned. Respondents also felt they did a meaningful job, as they were *‘working for the greater good*’ and were ‘*glad to be able to do something*’. They further experienced a great amount of openness and respect from their colleagues, as one respondent said that *‘everyone could show his or her best side and as such learn and benefit from each other’*.

Positive experiences were further related to the ‘decision-making’ and ‘work processes’. For instance, participants experienced less bureaucracy and appreciated how fast decisions were made: *‘Suddenly, much was possible in a short space of time that would otherwise have remained on the shelf for a long time’,* Lastly, a few respondents answered *‘nothing’* to the question on what positive things they would like to keep in the future.

### Lessons learned

**Fig. 2:**
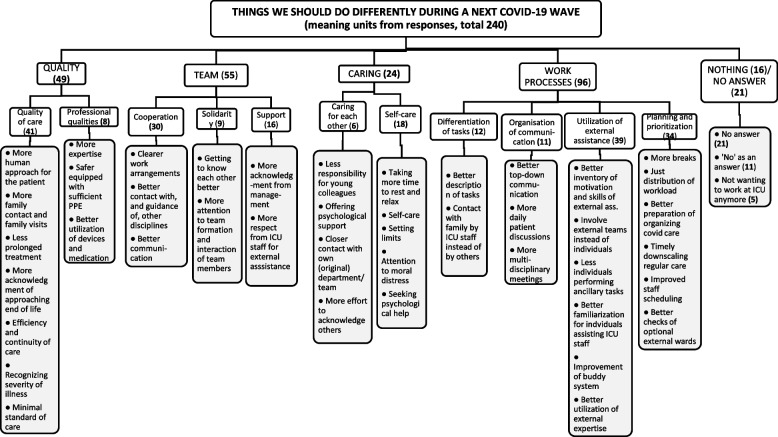
Categorization of answers to the open question ‘*What things should we do differently during a next COVID-19 wave?*’. This Framework shows the overall clusters (upper level), themes and codes (last level) that emerged from analyzing the second open-ended question. The number of meaning units (total 240) is mentioned between brackets for every cluster. ICU = Intensive Care Unit; PPE = Personal Protective Equipment

An overview of things that respondents would now do differently in a similar situation (such as another COVID-19 wave) is presented in Fig. 2. These things can be seen as lessons learned and relate to the categories ‘quality’, ‘team, ‘caring’ and ‘work processes’. Especially the latter category on ‘work processes’ covered the large majority of responses, followed by the categories ‘quality’ and ‘team’.

Comparable to the positive experiences, several responses again related to ‘quality’, with subcategories ‘quality of care’ and ‘professional qualities’, but now more prominent. According to some, the quality of care was at risk by a lack of continuity of care due to a shortage of personnel; it was therefore suggested to *‘create more continuity in the care system’*. It was also emphasized by many respondents that treatment should not be continued too long, hence, one suggested that ‘*the current experiences about worse prognostic signs should be better translated into a limited duration of treatment’*, and another said that ‘*decisions to stop or restrict treatment should be defined *a priori* and more clearly, and must be complied to’*. This finding thereby confirms the quantitative responses. Other suggestions in this cluster related to the communication with patients and family, as many said that family should be more involved and allowed to visit patients, and one respondent advised to ‘*find ways to better show our human dimension: open masks so that your face can be seen better and your voice can be heard more clearly’* and to also give patients more dignity, for instance by putting *‘personal belongings and stories around their beds’*.

Considering the professional qualities: this was now more about one’s own qualities rather than those of colleagues. For instance, one respondent wrote: *‘I did not feel sufficiently competent to supervise a whole unit’*.

Other lessons mainly focused on the cluster ‘team’. Although the external helpers were greatly appreciated, there were also several respondents who did not share this feeling, as one felt *‘treated as if we were incapable of doing anything’*. More attention for developing a coherent team feeling was therefore suggested. Some respondents also missed the presence of certain disciplines or they felt a lack of communication: *‘there should be more room for discussion between supervisor and fellow physicians’* and* ‘nurses should also be more involved in treatment decisions*’. Many respondents therefore suggested that there should be more evaluation moments, with all staff members and separately with their respective teams.

A new cluster of lessons learned related to ‘caring’ emerged, both for oneself and for each other. Several respondents said that they would take *‘more time for rest and leisure’* during a next COVID-wave, and to better set personal boundaries: ‘*to take better care of myself, as I am too quickly inclined to say that I’m fine’*. Seeking and offering psychosocial support within their own team and at more appropriate times was also mentioned. Some said that they would give fewer responsibilities to young and inexperienced staff in a subsequent wave, and they would try to reduce workload. For instance, one respondent intended to *‘save my personnel: not one ICU nurse taking care of four patients, especially if it is a newly graduated nurse’*.

Lastly, most suggestions were made related to ‘work processes’: better task differentiation, more consultation moments, more breaks, better deployment of non-ICU staff and better prioritization of tasks.

## Discussion

This study further confirms the high levels of moral distress experienced by ICU staff and the many moral challenges around end-of-life care at the frontline during the COVID-19 pandemic, and shows that these experiences clearly differed between nurses and physicians. Yet, also positive experiences and lessons learned were mentioned.

### Moral challenges and distress

Considering moral challenges, our findings demonstrate that ICU staff experienced several challenges relating to situations in which quality of care was perceived to be compromised, or uncertainty or distress was felt around EOL decisions. Most respondents felt that they were less able to provide the same quality of patient care as usual. This finding was also found in another recent Dutch study among healthcare professionals (from a variety of backgrounds in healthcare) on end-of-life care during covid times, where almost half of the respondents felt that EOL care had been limited because of the COVID-19 crisis [8].

This study further shows the negative impact of visitor restrictions on nurses’ perceived ability to provide good care, which confirms the emphasis from our respondents on the problematic limitations in visiting possibilities for patients’ relatives. The feeling of not being able to provide adequate quality of care resulted in moral distress, particularly in emotional stress, worrying about work and less willingness to go to work. This moral distress might have been exacerbated by both the feeling that medical interventions were sometimes started even though they would probably only prolong the suffering of a patient, and the feeling that EOL decisions were postponed too often or for too long. It is important to note, however, that if no one felt there was delay or postponement, it would likely be the other way around (i.e. decisions about withholding or withdrawing treatment were made too quickly and expeditiously).

However, suggesting a causal link between experiencing moral challenges and moral distress is complicated, as reported in the review by Schofield and colleagues [20]. Moral challenges are an inherent part of working at the ICU and they do not always lead to moral distress. But often moral distress is related to experiencing severe and frequent moral challenges and can be seen as a symptom or ‘after-effect’ of having to handle moral challenges [22].

Despite the signs of moral distress, the team cooperation was well appreciated: most healthcare professionals felt respected in their work and thought their colleagues provided good patient care. Results also indicated that different opinions and values regarding EOL care were valued and accepted; moral challenges could be discussed openly. These findings seem to indicate a relatively safe ethical climate, which is also essential for preventing and diminishing moral distress, as has been suggested before [12].

### Differences between nurses and physicians

During the current COVID-19 crisis, half of the nursing staff in this study showed signs of moral distress whilst working at the ICU, which was significantly more than physicians. Even during the first wave of COVID-19, concerns were expressed about the moral burden on nurses as the most heavily affected frontline healthcare workforce [11]. Another study [46] examined the sources for moral distress in ICU nurses in Canada by inviting nurses to describe critical incidents, and found that moral distress was especially induced by feelings of powerlessness: when being confronted with limited resources, patients dying without their loved ones and their perceived limited influence on treatment decision-making processes. The latter finding was also shown in the study among Spanish critical care nurses [28], who found that ethical conflict was especially experienced when feeling insufficiently involved in the decision-making process and watching a patient suffering. This might to some extent explain the differences between nurses and physicians, as physicians might have had more influence on decision-making processes and, hence, might have felt less powerless during the pandemic compared to nurses.

Interdisciplinary differences in experienced moral distress have already been demonstrated prior to the COVID-19 crisis [26, 27, 47, 48], and given the cross-sectional nature of this study, it is difficult to properly discern how much of the experienced moral distress (and the differences between disciplines) was related to the COVID-19 crisis, and how much was pre-existing. However, the respondents themselves reported a marked reduction in enjoyment of their work compared to before the COVID-19 crisis, indicating that at least a significant part of the experienced moral distress was caused by the current situation. Donkers et al. [49] compared levels of moral distress of Dutch ICU staff during the COVID-19 crisis with those from a pre-COVID-19 control group and found that both nurses and intensivists reported significant higher levels of moral distress during the COVID-19 crisis, but that these differences between these professions were smaller during the pandemic. The positively appreciated ethical climate in our study might partly explain this.

### Ideas for improvement

This study is one of the first studies to also look at the positive lessons learned by healthcare professionals from this period; it highlights constructive suggestions for improving the quality and organization of care. The importance of accessible support services to address moral distress was also suggested in another recent Dutch study on ICU staff’s moral distress [49]. Positive experiences and presence of, or opportunities for, strengthened moral resilience of healthcare professionals are often overlooked in studies regarding moral distress and/or COVID-19. Usually, studies on moral distress mainly stress its negative aspects, while experiencing moral challenges and moral distress are also signs of potential venues in which one can improve the moral quality of (the organisation of) care.

Lamiani and colleagues [12] described four responses to moral distress by critical care physicians: avoidance, acquiescence, resistance and reinterpretation, of which the last one refers to finding ‘new possible ways to be good physicians under challenging situations’, thereby restoring their moral integrity and enhancing moral resilience, but also improving quality of care. The positive experiences of ICU staff in this study highlight this fourth fact as well, in the sense that, even in a pandemic crisis, ICU workers were able to demonstrate high flexibility at various levels. This was also shown in a recent study on moral distress of critical care physicians during the first COVID-19 wave in Italy, where some physicians described how they were able to adapt to the harsh reality and find creative ways to regain their sense of being a good doctor [12]. Yet, we need further research in order to find out whether these positive experiences and flexibility remained the same during subsequent COVID-19 waves.

### Directions for ethics support

Since the COVID-19 crisis is not yet over, it is likely that ICU staff still experience the reported moral distress and thus there is a significant risk that they will develop (or are already developing) chronic moral distress, leading to moral injury and psychological trauma, something that recent studies have also warned against [12, 25]. Our study shows that ICU staff expressed a need for more attention to both quality of care and self-care during a subsequent wave. This information helps to define and tailor ethics support services to ICU staff in potential future waves of the COVID-19 pandemic or comparable challenging situations.

Institutional ethics support services were already in place during the period studied (e.g. a 24/7 ethics support telephone hotline) but could perhaps have been adjusted by more explicitly considering feelings of moral distress and how to deal with them. Also, more innovative and less time-consuming ways of providing ethical support in and during work processes [50], such as using a moral compass for specific moral themes [51] or the CURA instrument for low-threshold ethical reflection [52, 53], could be applied. For this, an empirical-ethical study among ICU staff would be helpful to analyze experiences of and ways of dealing with morally distressing situations, in order to tailor future ethics support tools to their needs. In designing these innovative and thematic oriented forms of ethics support related to moral distress, various conceptualizations of moral distress should also be taken into account [54]. Ethics support services in general have been shown to be helpful in dealing with moral distress, restoring moral resilience [14, 29] and caring for one’s self and others [2, 31], also during COVID-19 care [55].

The findings of this study form the basis for further in-depth dialogues and focus group interviews with the ICU staff involved to improve the quality and organization of care, on the one hand, and embed innovative ways of providing ethics support services, on the other. For instance, Kok and colleagues [25] have recently recommended healthcare organizations to ‘stimulate grassroots dialogues on moral requirements in pandemic times’, such as organizing moral case deliberations. In order to stress the relevance and shared ownership of our study findings, we presented the findings of this study to the ICU staff at two team meetings. Especially the positive experiences and the things that can be improved in the future can be useful for the ICU teams that participated in this study. After the presentations, we also shared a Dutch summary of the main results and lessons learned with all respondents. In this way, results from scientific studies were made relevant for direct improvements during ongoing times of crisis. Currently, plans are made to provide ethics support by structural moral case deliberations at the ICU units.

### Strengths and limitations

A main strength of our study is the complementary combination of quantitative and qualitative methods, as respondents’ answers to the open-ended questions confirmed and clarified their scores on the closed questions. Furthermore, in focusing on the positive aspects and lessons learned, our study takes an extra step and has added value compared to the existing literature on moral distress during pandemic times.

One of the limitations lies in the fact that not all respondents worked at the ICU for throughout the first wave. Moreover, during the first COVID-19 wave, constant adjustments were made to both the quality and organisation of care. This means that different answers probably refer to different moments in time and different working conditions during the first wave. Furthermore, there was a relatively large amount of healthcare professionals who did not fill-out the questionnaire, as often experienced in studies where questionnaires are sent via email [56, 57]. It is therefore not certain to which extent our respondents in this cross-sectional survey during a crisis period form a representative group of ICU workers during COVID-times. Future research might compare the various studies on this topic to shape a more general and representative picture, for instance by means of an overall scoping review.

With respect to the estimated response rate: it was not possible to receive all the individual email addresses of the external staff, so some automated mailing lists were used. This caused a relative underestimation of the percentage of staff that completed the questionnaire.

The questionnaire used in this study consists of questions from three existing scales, not all of which have been validated. Because of the urgency and relevance, the authors decided to create this composite questionnaire, to eventually contribute to the further validation of these complex phenomena. For that reason, we performed a quality check on our predefined clusters of items with inter-item correlations, which showed acceptable between items associations. Lastly, it should be noted that the positive experiences and lessons learned were collected via open-ended questions and hence, lack contextual information and conceptual depth.

## Conclusions

This study shows that ICU staff experienced moral distress notwithstanding a relatively positive ethical climate during the first wave of the COVID-19 pandemic, and experienced several moral challenges (among others regarding EOL decisions). As other studies corroborate, nurses experienced significant more moral distress than physicians did. In a unique way, this study also reported on the positive experiences and lessons learned, such as personalizing and prioritizing communication with patients, relatives and among care professionals, and investing in both self-care as well as competences of staff. This sheds light on improvements in practice and on how to design tailor-made forms of ethics support, by fostering low-threshold opportunities to reflect on morally challenging situations, restoring moral resilience and realizing room for self-care and empowering team spirit. Further research is needed to identify ways in which existing and new clinical ethical support services can support ICU staff during and beyond this exceptional COVID-19 pandemic and provide recovery and reflection in both current and future stressful times.

## Supplementary Information

**Additional file 1.** Details about the questionnaires underlying the survey questions [58–60].

## Data Availability

The datasets are available from the corresponding author upon reasonable request.

## Abbreviations

ICU: Intensive Care Unit
EDMCQ: Ethical Decision-Making Climate Questionnaire
EOL: End of Life
MDS-R: Moral Distress Scale Revised
STROBE: Strengthening the Reporting of Observational Studies in Epidemiology

## Footnotes

1 Abbreviations of authors’ names: MvZ = Mark van Zuylen, JS = Janine de Snoo-Trimp, BM = Bert Molewijk.
