# Supplementary material for: Moral distress and positive experiences of ICU staff during the COVID-19 pandemic: lessons learned
Source: BMC Med Ethics. 2023 Jun 8;24:40. doi: 10.1186/s12910-023-00919-8 (PMC10249541; doi:10.1186/s12910-023-00919-8)
Supplement: Supplementary file 1 — Additional file 1. Details about the questionnaires underlying the survey questions [58–60]. [file 12910_2023_919_MOESM1_ESM.docx]

**Supplementary file 1** – **Details about (and reasons for) the questionnaires underlying the survey questions**

The Moral Distress Scale-Revised (MDS-R) consists of 21 items and is intended for ICU nursing staff (34). It is derived from the original MDS questionnaire (36). It uses a 4-point Likert scale for describing the frequency the statement from the question is observed and the intensity of feelings this disturbance produces. The composite score ranges from 0–336. The MDS-R has good content validity and reliability (34).
The 32-item EDMCQ (33) is an easy-administrable questionnaire measuring ethical decision making. It covers perceptions regarding key domains of ethical decision-making: awareness of specific patient situations and consequent interdisciplinary discussion, speaking up whilst maintaining a safe environment and motivating ethical awareness in multidisciplinary teams by sharing views, morals and ideas. Questions are subdivided into 7 sections: self-reflective and empowering leadership by doctors, practice and culture of open interdisciplinary reflection, culture of not avoiding EOL decisions, culture of mutual respect within the interdisciplinary team, active involvement of nurses in EOL care and decision-making, active decision-making by doctors, practice and culture of ethical awareness. It uses a 4 or 5-point Likert scale and has good factorial validity (33).
The Individual Detection and Reflection Tool for Moral Stress (35) is an instrument in Dutch, developed with the support of the European Social Fund and the Flemish Government. It aims to provide healthcare professionals with more insights into their experiences of moral stress and consists of several short questionnaires. The first questionnaire investigates the extent to which certain characteristics of moral stress are present in the healthcare professionals themselves. A second questionnaire investigates the extent to which they experience negative effects of moral stress. A third test examines moral sensitivity. The fourth and fifth test gives insight into some risk-increasing and risk-reducing factors. It combines 7-point Likert scales with multiple answer multiple choice questions and open-ended questions.
The authors chose not to use The Ethical Climate Questionnaire (58-59) because we wanted to use fewer items and look more specifically focused on the ICU and end of life decision making. Similarly, the Hospital Ethical Climate Survey (60) was deemed too general and too extensive to use.
